# Early life nutrition influences susceptibility to chronic inflammatory colitis in later life

**DOI:** 10.1038/s41598-019-54308-6

**Published:** 2019-12-02

**Authors:** Delphine Ley, Jean-Luc Desseyn, Valérie Gouyer, Ségolène Plet, Sebastian Tims, Ingrid Renes, Mona Mischke, Frédéric Gottrand

**Affiliations:** 1 Univ. Lille, Inserm, CHU Lille, LIRIC UMR 995, F-59000 Lille, France; 2 Danone Nutricia Research, Utrecht, The Netherlands

**Keywords:** Disease model, Inflammatory bowel disease

## Abstract

The first thousand days of life are a critical time of development in humans during which the risk profile for diseases in later life can be modified. Nevertheless, long-term consequences of early environment on susceptibility to intestinal diseases have not yet been assessed. Using a mouse model of postnatal growth restriction (PNGR), we showed that early life nutrition influences intestinal maturation and gut health in later life. PNGR induced an alteration of the intestinal barrier in pups at weaning, resulting in increased intestinal permeability, and affected gut bacterial colonization. Specifically, pups with PNGR harbored a decreased bacterial diversity, higher *Enterococcus* spp., *Staphylococcus* spp., and *Escherichia-Shigella* spp., and lower *Odoribacter* spp. and several members of the Lachnospiraceae family. The lack of an efficient intestinal barrier in early life and the dysbiosis induced by PNGR were associated with a higher susceptibility to chronic colitis in adulthood.

## Introduction

The early life period, from conception until two years of age – the so-called first 1000 days of life – is a critical window during which the environment has a profound influence on the development of the fetus and infant and the risk profile for disease in later life^1^. While the concept of perinatal programming, or developmental origins of health and disease (DOHaD), has emerged as an important component of the pathogenesis of the metabolic syndrome and cardiovascular diseases in adulthood^2,3^, the impact of the early life environment on gut health in later life is still largely unknown.

The intestinal barrier consists of the intestinal microbiota, the mucus layer, the intestinal epithelium and its intercellular junctions, and the intestinal immune system^4^. Maturation of the intestinal barrier starts during the first trimester of pregnancy in humans and continues after birth until three years of age^5^. The epithelial barrier and the intestinal microbiota develop in a mutually beneficial relationship. The intestinal epithelium and the mucus layer provide to the microbiota a niche with the nutrients necessary for bacterial growth. In turn, the intestinal microbiota is critical for the maturation and the maintenance of the epithelial barrier, by promoting epithelial cell proliferation, maintenance of tight junctions, and mucus production^6^. The epithelial barrier and the microbiota contribute together during the early postnatal period to the education of the immune system and the acquisition of immune tolerance to commensal bacteria and dietary antigens^7,8^.

The integrity of the intestinal barrier is essential for immune homeostasis. Dysfunction of the epithelial barrier, associated with dysbiosis (imbalance in normal intestinal microbiota), is implicated as a critical component of chronic inflammatory disorders, such as obesity, food allergy, and chronic intestinal disorders including irritable bowel syndrome, inflammatory bowel disease and celiac disease^4,5^. Continuous exposure of the immune system to antigens from the intestinal lumen, due to increased intestinal permeability, could result in the development and maintenance of a chronic inflammatory response as has been observed in these disorders.

Increasing evidence suggests that the perinatal environment can affect the developing intestine, including the intestinal microbiota, and modify the risk profile for chronic intestinal disorders in later life: (i) Epidemiological studies have highlighted the link between early life environment and later susceptibility for chronic inflammatory intestinal disorders; (ii) Early life environmental factors, including mode of delivery, early-life nutrition, and antibiotic use, play critical roles in the development of the microbiota composition; (iii) Animal models have shown that early life environment affects the development of the gastrointestinal tract (for review see Ley *et al*.^9^). However, only few studies have investigated the long-term consequences of the perinatal environment on susceptibility to chronic intestinal disorders^10–13^. Further experimental studies are needed to confirm the impact of early life environment on gut health in later life and identify the underlying mechanisms.

Using a mouse model of postnatal growth restriction (PNGR), we investigated the programming effect of early life nutrition on gut function and resilience in later life. We hypothesized that PNGR could adversely alter the intestinal maturation state and bacterial colonization in pups in early life, thereby leading to an increased susceptibility to chronic colitis in adulthood.

## Results

### Pup growth and body composition

**Figure 1:**
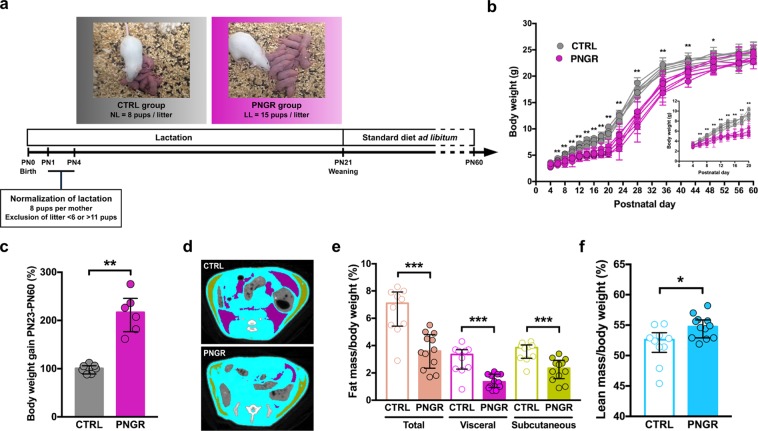
Pup growth and body composition were affected by PNGR. **(a**) Experimental design. **(b**) Body weight curves and **(c**) body weight gain after weaning in control (CTRL) (n = 7 litters) and postnatal growth restriction (PNGR) (n = 6 litters) groups. **(d**) Body composition (purple = visceral fat mass; green = subcutaneous fat mass; blue = lean mass), **(e**) fat mass and **(f**) lean mass in adult mice with PNGR (n = 12 from 5 litters) and CTRL mice (n = 10 from 3 litters) assessed with computed tomography scanner. Results are representative of 3 independent experiments. *P < 0.05; **P < 0.01; ***P < 0.001.

To investigate the impact of early life nutrition on gut maturation and functionality, we used a mouse model of PNGR induced by undernutrition during the suckling period by increasing the litter size (Fig. 1a). Because the gut matures during the postnatal period in a relatively short time^14^, neonatal rodent models are valuable to study the influence of early life nutrition on intestinal maturation and related intestinal disorders.

Pups were randomly attributed at postnatal day (PN) 4 to either control (CTRL) litter (8 pups) or large litter (LL, 15 pups). Body weight was similar in all pups at PN4. Pups from LL displayed a significant lower body weight from PN6 and during the entire early postnatal period (*P*-values range between PN6 and PN21 = 0.001–0.004) (Fig. 1b). The body weight of pups with PNGR was lower than the 10^th^ percentile body weight distribution in the CTRL group from PN6 to PN21. After weaning, pups with PNGR showed a catch-up growth outlined by higher body weight gain (Fig. 1c), thereby reaching a normal body weight in adulthood (Fig. 1b). Despite normal body weight in adulthood, the body composition measured by CT-Scan was different between PNGR and CTRL groups (Fig. 1d). Adults from PNGR displayed lower total, visceral and subcutaneous fat mass (*P = *0.0003, *P = *0.0004, and *P = *0.0008, respectively, Fig. 1e), whereas lean mass was higher (*P = *0.016, Fig. 1f).

### PNGR alters intestinal maturation

In order to investigate the impact of PNGR on intestinal maturation, we examined intestinal morphogenesis and functionality in ileum and colon in pups at weaning (PN21).

#### PNGR alters postnatal intestinal morphogenesis

**Figure 2:**
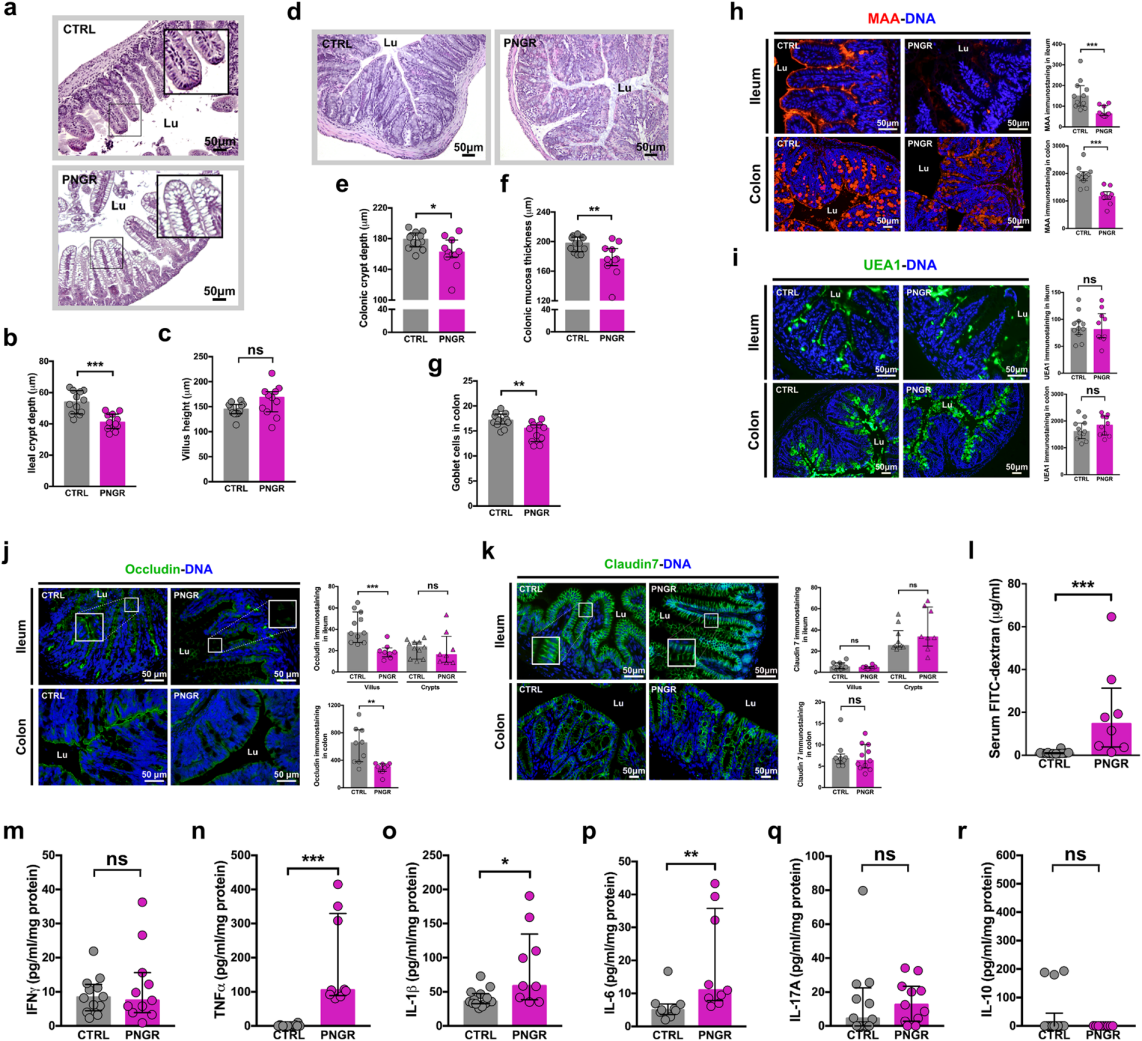
PNGR altered intestinal maturation in pups at weaning. **(a**) Representative haematoxylin and eosin (HE) staining of ileum sections from control (CTRL) and postnatal growth restriction (PNGR) pups. **(b**) Crypt depth and **(c**) villus height in ileum at postnatal day 21 (PN21) in CTRL (n = 12 from 4 litters) and PNGR (n = 11 from 4 litters) groups. **(d**) Representative HE staining of colonic sections from CTRL and PNGR pups. **(e**) Crypt depth, **(f**) mucosa thickness, and **(g**) number of goblet cells in colon at PN21 in CTRL (n = 12 from 4 litters) and PNGR (n = 11 from 4 litters) groups. Representative staining of **(h**) sialylated glycans by MAA lectin and **(i**) fucosylated glycans by UEA1 lectin in ileum and colon at PN21 in CTRL (n = 11 from 4 litters) and PNGR (n = 10 from 4 litters) groups. **(j**) Representative immunostaining of occludin in ileum and colon at PN21 in CTRL (n = 9–11 from 4 litters) and PNGR (n = 8–9 from 4 litters) groups. **(k**) Representative immunostaining of claudin7 in ileum and colon at PN21 in CTRL (n = 9 from 4 litters) and PNGR (n = 11 from 4 litters) groups. **(l**) *In vivo* intestinal paracellular permeability to 4 kDa FITC-dextran at PN21 in CTRL (n = 12 from 4 litters) and PNGR (n = 8 from 4 litters) groups. **(m–r**) Cytokines expression in colon at PN21 in CTRL (n = 9–14 from 4 litters) and PNGR (n = 9–11 from 4 litters) groups. Results are representative of 2–3 independent experiments. *P < 0.05; **P < 0.01; ***P < 0.001.

PNGR was associated with an immature ileal epithelium at PN21, characterized by the presence of vacuolated villus enterocytes (Fig. 2a) that normally disappear during the third week of life in rodents. Ileal crypt depth was lower in pups with PNGR (*P* = 0.0005, Fig. 2b). There was not difference in ileal villus height (*P* = 0.08, Fig. 2c). Colonic crypt depth was lower (*P* = 0.04, Fig. 2d,e), as well as mucosa thickness (*P* = 0.004, Fig. 2d,f), in pups with PNGR at PN21. The number of goblets cells per colonic crypt was decreased in pups with PNGR (*P* = 0.004, Fig. 2g).

#### PNGR disturbs brush border enzymes expression in pups at weaning

To examine the ileal digestive and detoxifying capacity, the expression pattern and activity level of the major brush border enzymes dipeptidyl peptidase IV (DPPIV) and intestinal alkaline phosphatase (IAP) were analyzed. DPPIV and IAP expression at the brush border seemed to be reduced in the ileum of pups with PNGR at PN21 as revealed by immunohistochemistry (*P* < 0.0001 and *P* = 0.0003, respectively, Suppl. Fig. S1a). However, DPPIV was expressed in the cytoplasm of vacuolated villus enterocytes of pups with PNGR (Suppl. Fig. S1a). DPPIV activity level in ileum was similar in both groups at PN21 (*P* = 0.95, Suppl. Fig. S1b). In contrast, IAP activity level in the ileum was increased by 2.6 times in pups with PNGR at PN21 (*P* = 0.04, Suppl. Fig. S1c). The higher IAP activity in PNGR group could be a compensatory effect in order to increase LPS detoxifying capacity^15^.

#### PNGR induces changes in the glycosylation pattern of the intestinal epithelium in pups at weaning

Glycosylation of the host tissues is modulated by diet and microbiota. In turn, the intestinal glycome can modulate microbiota composition by providing nutrients that promote the growth of specific bacteria^16,17^. In order to determine if PNGR may impact the *O*-glycosylation, we studied by immunofluorescence sialylated and fucosylated glycans expression, assessed by immunostaining at PN21 with *Maackia amurensis* agglutinin (MAA) and *Ulex europaeus* agglutinin (UEA1) lectins, respectively. MAA staining was lower in ileum and colon of pups with PNGR (*P* = 0.0005 and *P* < 0.0001, respectively, Fig. 2h) while no difference between the two groups was found using UEA1 (*P* = 0.97 and *P* = 0.47, respectively, Fig. 2i).

#### PNGR alters intestinal barrier in pups at weaning

To evaluate the impact of PNGR on epithelial barrier function, we measured by immunofluorescent staining on ileum and colon sections the expression of the two major tight junction proteins claudin7 and occludin. PNGR was associated with an impaired expression of tight junction proteins in both tissues at PN21. The expression of occludin was limited to the crypts in the ileum of pups with PNGR (Fig. 2j), whereas in CTRL pups occludin staining was seen in crypts and along the villi. In colon, occludin was less expressed in pups with PNGR compared to CTRLs (*P* = 0.001, Fig. 2j). Quantification of Claudin7 staining was similar in the ileal crypts and villi in both groups. However, claudin7 expression was distorted in the villus epithelium of pups with PNGR, where claudin7 was mostly expressed on the basolateral side of enterocytes (Fig. 2k). In colon, claudin7 distribution seemed similar in both groups (*P* = 0.75, Fig. 2k). To assess whether the abnormal expression pattern of tight junction proteins has an impact on intestinal barrier integrity, we next measured *in vivo* the intestinal permeability to 4 kDa FITC-labelled dextran at PN21. Pups with PNGR showed an increased intestinal paracellular permeability (*P* < 0.0001, Fig. 2l).

#### PNGR induces an increase in pro-inflammatory cytokines expression in pups at weaning

To examine whether the increased intestinal permeability was associated with a pro-inflammatory response of the mucosal immune system, we measured several cytokine levels in ileal and colonic samples. IFNγ level was similar in both groups in the colon at PN21 (*P* = 0.98, Fig. 2m). The expression at the protein level of the pro-inflammatory cytokines TNF-α (*P* < 0.0001, Fig. 2n), interleukin (IL) -1β (*P* = 0.01, Fig. 2o), and IL-6 (*P* = 0.003, Fig. 2p), was increased in the colon in pups with PNGR. The expression of the cytokines associated with Th17 (IL-17) and regulatory (IL-10) immune responses was similar in both groups (*P* = 0.44, Fig. 2q and *P* = 0.35, Fig. 2r, respectively). In ileum, the expression of TNF-α was higher in pups with PNGR (*P* < 0.0001, Suppl. Fig. S2). IFN-γ, IL-1β, IL-17A, IL-6 and IL-10 levels were similar in both groups (Suppl. Fig. S2).

Taken together, these data show that PNGR adversely altered the intestinal maturation status and the integrity of the intestinal barrier, which was associated with a pro-inflammatory status within the intestinal mucosa in early life.

### PNGR induces changes in intestinal functions in adult mice

To examine whether alterations found in the intestinal tissues of pups with PNGR may impact the adulthood, intestinal morphology and functions were investigated in ileum and colon in adult mice at PN60.

**Figure 3:**
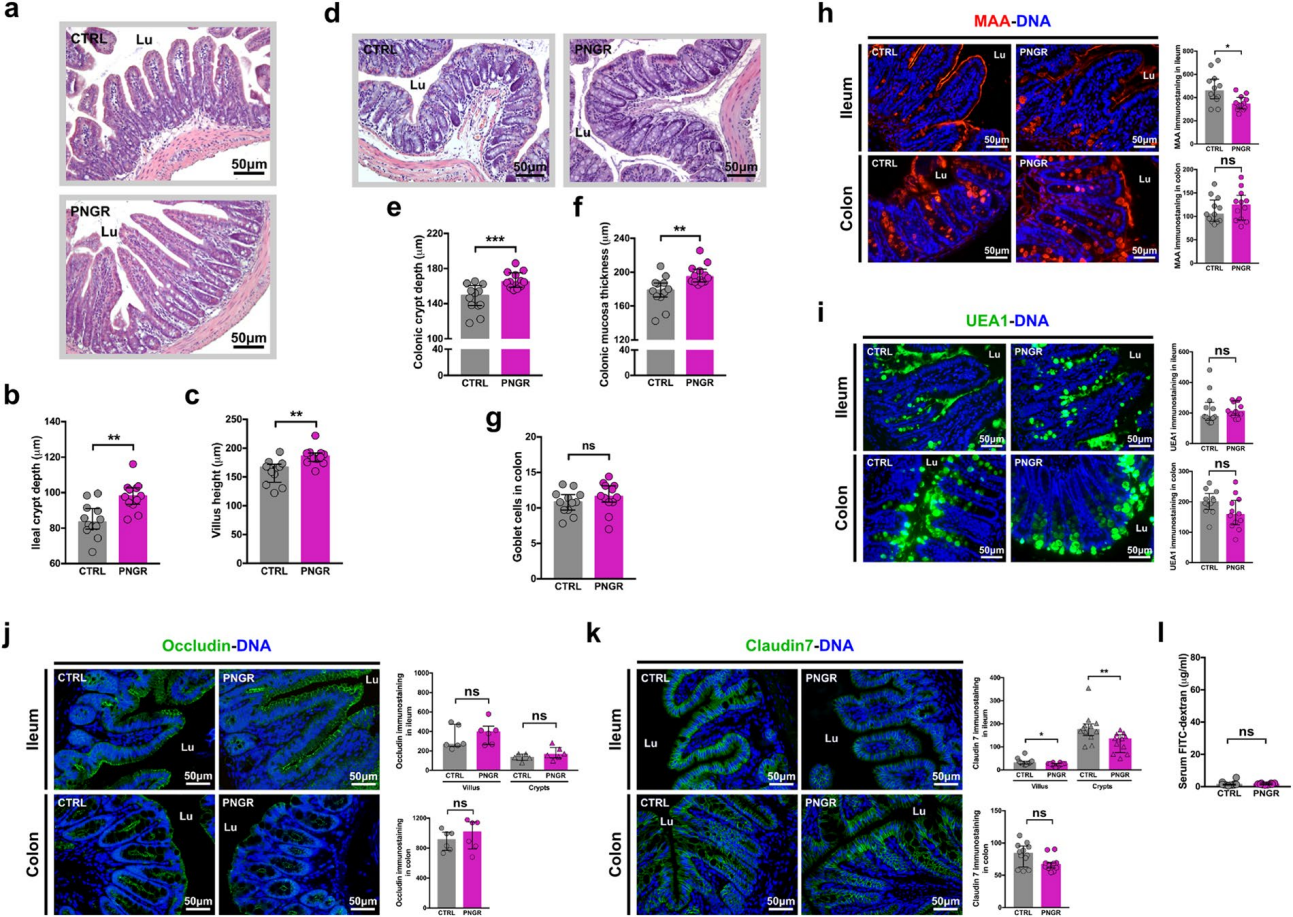
PNGR had long-term impact on gut health status in adult mice. **(a**) Representative haematoxylin and eosin (HE) staining of ileum sections from control (CTRL) and postnatal growth restriction (PNGR) adult mice. **(b**) Crypt depth and **(c**) villus height in ileum at postnatal day 60 (PN60) in CTRL (n = 12 from 4 litters) and PNGR (n = 12 from 4 litters) groups. **(d**) Representative HE staining of colonic sections from CTRL and PNGR adult mice. **(e**) Crypt depth, **(f**) mucosa thickness and **(g**) number of goblet cells in colon at PN60 in CTRL (n = 12 from 4 litters) and PNGR (n = 12 from 4 litters) groups. Representative staining of **(h**) sialylated glycans by MAA lectin and **(i**) fucosylated glycans by UEA1 lectin in ileum and colon at PN60 in CTRL (n = 12 from 4 litters) and PNGR (n = 12 from 4 litters) groups. **(j**) Representative immunostaining of occludin in ileum and colon at PN60 in CTRL (n = 6 from 3 litters) and PNGR (n = 6 from 3 litters) groups. **(k**) Representative immunostaining of claudin7 in ileum and colon at PN60 in CTRL (n = 12 from 4 litters) and PNGR (n = 12 from 4 litters) groups. **(l**) *In vivo* intestinal paracellular permeability to 4 kDa FITC-dextran at PN60 in CTRL (n = 12 from 4 litters) and PNGR (n = 10 from 4 litters) groups. Results are representative of 2–3 independent experiments. *P < 0.05; **P < 0.01; ***P < 0.001.

Ileal crypt depth (*P* = 0.002, Fig. 3a,b) and villus height (*P* = 0.001, Fig. 3a,c) were increased in adult mice with PNGR compared to adult CTRL mice. Similarly, in colon, crypt depth (*P = *0.0005, Fig. 3d,e) and mucosa thickness (*P* = 0.002, Fig. 3d,f) were increased in adult PNGR mice in comparison with adult CTRL mice. Further, the number of goblet cells in colon was not different in both groups (*P* = 0.25, Fig. 3g).

The expression of DPPIV and IAP protein at the brush border in ileum was similar in both groups at PN60 (*P* = 0.24 and *P* = 0.35, respectively, Suppl. Fig. S3a). Yet at the enzyme activity level, DPPIV as well as IAP were decreased by 1.4 times in adult ileum with PNGR (*P* = 0.003 and *P* = 0.02, respectively, Suppl. Fig. S3b and S3c) compared with adult CTRL mice.

Immunostaining with MAA lectin to assess sialylation showed less sialylated glycans in the ileum of adult mice with PNGR compared to CTRLs (*P* = 0.02, Fig. 3h). Fucosylated glycans assessed by immunostaining with UEA1 lectin was similar in both groups in ileum and colon at PN60 (*P* = 0.35 and *P* = 0.07, respectively, Fig. 3i).

Concerning the epithelial barrier integrity, the expression of occludin was not different in ileum neither in the colon between adult PNGR and CTRL groups at PN60 (*P* = 0.25 and *P* = 0.48, respectively, Fig. 3j). Claudin7 expression in ileum of adult PNGR mice was decreased along the whole crypt-villus compared to CTRL mice (*P* = 0.03 in villus and *P* = 0.005 in crypts, Fig. 3k) but not in the colon (*P* = 0.07, Fig. 3k). Finally, the intestinal paracellular permeability, measured four hours after orogastrical gavage of mice with 4 kDa FITC-labeled dextran solution was not increased in adult mice with PNGR compared to CTRLs (*P* = 0.23, Fig. 3l).

### PNGR alters gut bacterial colonization

**Figure 4:**
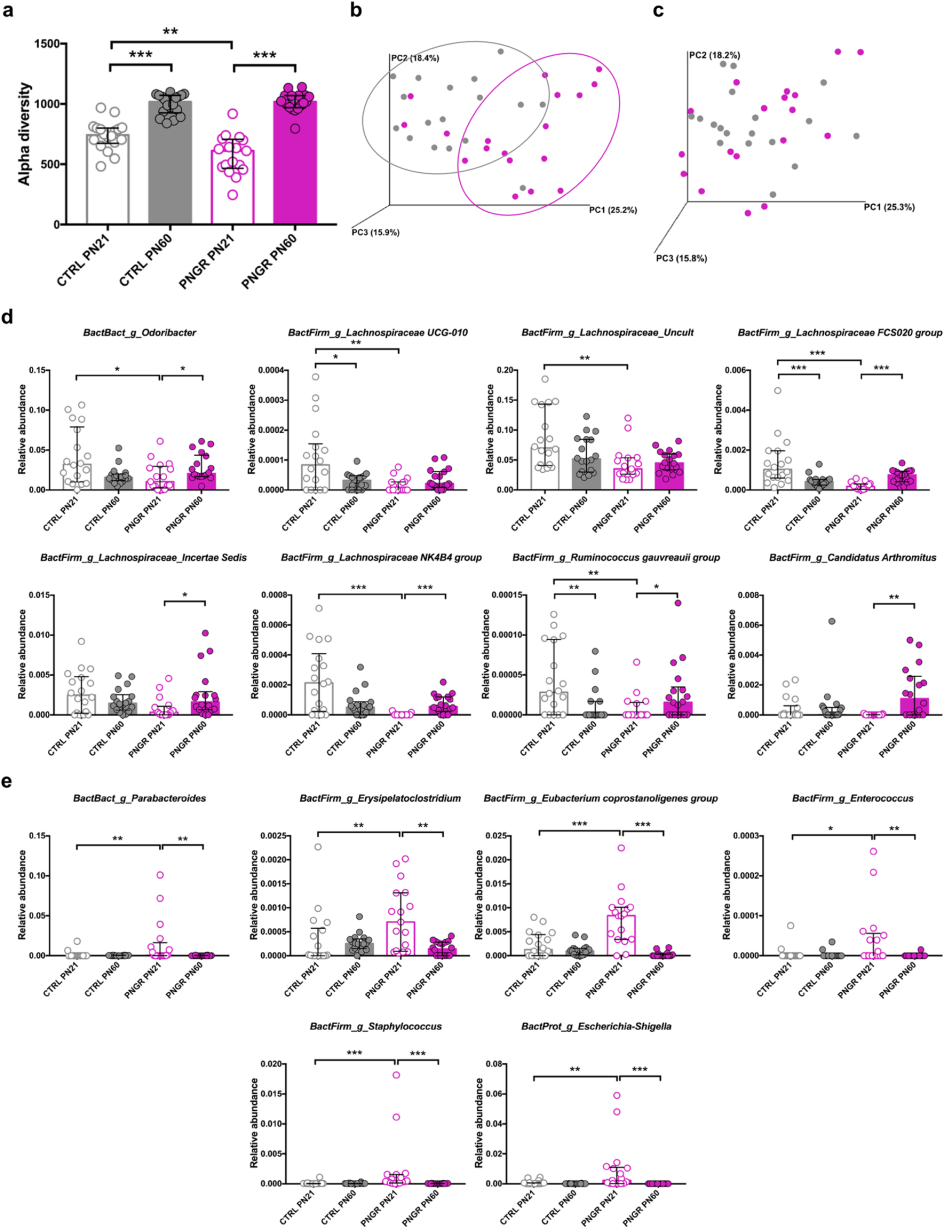
PNGR altered gut bacterial colonization. **(a**) α-diversity of fecal microbiota in control (CTRL, n = 18 from 6 litters) and postnatal growth restriction (PNGR, n = 17–18 from 6 litters) groups at postnatal day 21 (PN21) and postnatal day 60 (PN60). Phylogenetic distance between samples of fecal microbiota at **(b**) PN21 and **(c**) PN60 in CTRL and PNGR groups. Bacterial genera with **(d**) low abundance and with **(e**) high abundance at PN21 in pups with PNGR compared to CTRL. Results are representative of 3 independent experiments. *P < 0.05; **P < 0.01; ***P < 0.001.

Because the intestinal microbiota is critical for the maturation and defense of the epithelial barrier and the education of immune system, we studied microbiota richness and composition from fecal samples at PN21 and PN60 using bacterial 16S *rRNA* gene amplicon sequencing (Illumina MiSeq). The median number of read per sample was 66,397 (58,818–76,509), divided over 2,075 Operational Taxonomic Units (OTUs). The α-diversity showed an increase in microbiota richness between PN21 and PN60 in both groups (Fig. 4a) and, interestingly, a lower richness in pups with PNGR (*P* = 0.0014, Fig. 4a). At PN60, there was no difference anymore between the two groups (*P* = 0.70, Fig. 4a). The overall fecal microbiota composition as measured by weighted Unifrac distance showed a slight separation of groups along PC1 in a principal coordinate analysis (PCoA) at PN21 (Fig. 4b), suggesting a difference between the CTRL and PNGR groups. However, the PN60 samples were not separated in the PCoA plot and overlapped (Fig. 4c), suggesting that the fecal microbiota was more similar in adulthood. The differences found in the relative abundance levels of the genera were in agreement with a more distinct microbiota at PN21 as compared to PN60, as 21 genera were significantly different between the two study groups at PN21 and only one genera at PN60 (Table 1). Over time, thirty-two genera were developing in the same way in both groups between PN21 and PN60 (Table 1). Seven genera showed opposite development in time between the two groups. *Odoribacter* spp. and several Firmicutes members, mainly belonging to the Lachnospiraceae family, were lower at PN21 in PNGR compared to CTRL, and then increased towards PN60 in mice with PNGR (Fig. 4d). Several genera did not increase in the PNGR group between PN21 and PN60, whereas they increased in time in the CTRL group: *Atopobium* spp., several uncultured Ruminococcaceae family members (NK4A214 group, UCG-005, UCG-010, and UCG-013), *Turicibacter* spp., and *Bilophila* spp. (Table 1). *Candidatus Arthromitus* spp. increased from PN21 to PN60 in the PNGR group only (Fig. 4d), and Firmicutes Family XIII *Eubacterium nodatum* group as well as *Desulfovibrio* spp. (Proteobacteria) decreased in the PNGR group only (Table 1). Six genera were highly specific for PNGR at PN21 and showed high abundance levels in this group and time point only: *Parabacteroides* spp., *Erysipelatoclostridium* spp., *Eubacterium coprostanoligenes* spp., *Enterococcus* spp., *Staphylococcus* spp., and *Escherichia-Shigella* spp. (Fig. 4e). These findings demonstrate that PNGR affected early microbiota richness and composition in pups.

**Table 1:** Taxonomic profile of fecal microbiota.

Phylum	Genus	CTRL PN21 vs. PNGR PN21				CTRL PN60 vs. PNGR PN60				CTRL PN21 vs. CTRL PN60		PNGR PN21 vs. PNGR PN60		Description
		median PNGR	median CTRL	*p*	*q*	median PNGR	median CTRL	*p*	*q*	*p*	*q*	*p*	*q*	
Actinobacteria	*Atopobium*	0.0	0.0	0.50	0.26	0.0	0.0	0.22	0.36	**0.01**	**0.02**	0.44	0.17	No increase during time development in PNGR
Actinobacteria	*Coriobacteriaceae [Uncultured]*	6.18E-05	1.69E-05	0.06	0.07	0.00022	0.00035	0.36	0.44	**9.44E-06**	**7.82E-05**	**1.20E-05**	**2.95E-05**	Increase during time development^c^
Actinobacteria	*Enterorhabdus*	0.00068	0.00125	0.19	0.16	0.00501	0.00394	0.13	0.26	**2.40E-05**	**0.00015**	**8.82E-10**	**1.41E-08**	Increase during time development^c^
Bacteroidetes	*Bacteroidales S24-7 group [Other]*	0.0	0.0	0.32	0.21	3.35E-05	2.59E-05	0.92	0.70	**0.019**	**0.031**	**0.023**	**0.016**	Increase during time development^c^
Bacteroidetes	*Alistipes*	0.04430	0.03529	0.30	0.20	0.06374	0.04161	0.009	0.080	0.19	0.18	**0.035**	**0.022**	Increase during time development^b^
Bacteroidetes	*Bacteroidales S24-7 group [Uncultured]*	0.20150	0.25006	0.045	0.065	0.37349	0.39818	0.15	0.27	**1.54E-09**	**8.94E-08**	**2.24E-07**	**1.80E-06**	Increase during time development^c^
Bacteroidetes	*Bacteroides*	0.18501	0.08674	**0.0005**	**0.003**	0.03746	0.03175	0.25	0.40	**1.12E-07**	**2.16E-06**	**3.09E-09**	**3.30E-08**	Decrease during time development^c^; Remarkably high at PN21 in PNGR
Bacteroidetes	*Odoribacter*	0.01048	0.03219	**0.011**	**0.024**	0.02065	0.01583	0.013	0.079	0.059	0.075	**0.038**	**0.023**	Opposite time development
Bacteroidetes	*Parabacteroides*	0.00039	0.0	**0.003**	**0.011**	0.0	2.03E-05	0.40	0.46	0.28	0.24	**0.006**	**0.005**	Remarkably high at PN21 in PNGR
Bacteroidetes	*Porphyromonadaceae [Uncultured]*	0.0	0.0	0.63	0.30	4.04E-05	9.19E-05	0.052	0.15	**6.30E-06**	**6.09E-05**	**4.73E-06**	**1.38E-05**	Increase during time development^c^
Bacteroidetes	*Rikenellaceae RC9 gut group*	0.04054	0.00748	0.22	0.17	0.01054	0.00479	0.027	0.11	0.10	0.11	**0.038**	**0.023**	Decrease during time development^b^
Bacteroidetes	*Bacteroidales [Other]*	0.00010	0.00013	**0.014**	**0.028**	0.00073	0.00064	0.52	0.54	**2.14E-08**	**6.20E-07**	**7.99E-07**	**5.12E-06**	Increase during time development^c^; Repressed at PN21 in PNGR
Unclassified	*Bacteria [Other]*	1.22E-05	9.05E-05	0.035	0.055	0.00033	0.00042	0.21	0.36	**0.0002**	**0.0007**	**6.53E-05**	**0.0001**	Increase during time development^c^
Firmicutes	*Clostridiales vadinBB60 group [Other]*	4.89E-05	0.0	0.35	0.21	0.00150	0.00034	0.048	0.15	0.012	0.022	0.001	0.002	Increase during time development^c^
Firmicutes	*Lachnospiraceae [Other]*	0.00054	0.00356	**0.001**	**0.005**	0.00253	0.00297	0.32	0.44	0.63	0.43	**4.01E-06**	**1.29E-05**	Repressed at PN21 in PNGR
Firmicutes	*Anaerotruncus*	0.00483	0.00678	0.17	0.14	0.00209	0.00388	0.004	0.075	**0.003**	**0.007**	**0.002**	**0.002**	Decrease during time development^c^
Firmicutes	*Bacillus*	0.0	1.74E-05	**0.004**	**0.011**	0.0	0.0	0.36	0.44	**0.016**	**0.028**	0.57	0.20	Premature low level in PNGR
Firmicutes	*Candidatus Arthromitus*	0.0	0.0	0.25	0.18	0.00108	1.59E-05	0.098	0.22	0.65	0.43	**0.007**	**0.006**	Increase during time development in PNGR only
Firmicutes	*Clostridiales vadinBB60 group [Uncultured]*	0.00082	0.00110	0.66	0.31	0.00324	0.00368	0.96	0.71	**0.001**	**0.004**	**0.004**	**0.004**	Increase during time development^c^
Firmicutes	*Coprococcus 1*	0.00198	0.00206	0.81	0.35	0.00080	0.00092	0.41	0.46	**0.0007**	**0.003**	**0.013**	**0.011**	Decrease during time development^c^
Firmicutes	*Enterococcus*	0.0	0.0	**0.022**	**0.039**	0.0	0.0	0.53	0.54	0.98	0.58	**0.005**	**0.005**	Remarkably high at PN21 in PNGR
Firmicutes	*Erysipelatoclostridium*	0.00070	1.29E-05	**0.003**	**0.011**	0.00014	0.00025	0.043	0.15	0.16	0.15	**0.002**	**0.002**	Remarkably high at PN21 in PNGR
Firmicutes	*Erysipelotrichaceae [Uncultured]*	6.18E-05	0.00016	0.40	0.23	0.00075	0.00145	0.11	0.22	**0.006**	**0.012**	**0.025**	**0.016**	Increase during time development^c^
*Firmicutes*	*Eubacterium coprostanoligenes group*	0.00836	0.00127	**0.0004**	**0.003**	0.0	0.00010	0.002	0.057	0.69	0.45	**2.10E-06**	**9.61E-06**	Remarkably high at PN21 in PNGR
*Firmicutes*	*Eubacterium oxidoreducens group*	0.0	0.0	0.33	0.21	2.69E-05	1.64E-05	0.37	0.44	0.35	0.28	**0.0004**	**0.0006**	Increase during time development^b^
*Firmicutes*	*Intestinimonas*	0.00065	0.00012	**0.023**	**0.039**	0.00087	0.00100	0.52	0.54	**0.032**	**0.049**	0.68	0.23	Premature high level in PNGR
*Firmicutes*	*Lachnoclostridium*	0.00784	0.01151	0.17	0.14	0.00593	0.00545	0.61	0.56	**0.002**	**0.005**	0.13	0.064	Decrease during time development^a^
*Firmicutes*	*Lachnoclostridium 5*	2.45E-05	0.0	**0.0006**	**0.003**	0.0	8.49E-06	0.78	0.66	**0.002**	**0.006**	0.26	0.11	Premature high level in PNGR
*Firmicutes*	*Lachnospiraceae FCS020 group*	0.00018	0.00103	**9.02E-06**	**0.0004**	0.00075	0.00042	0.017	0.087	**0.0006**	**0.002**	**5.27E-05**	**0.0001**	Opposite time development
*Firmicutes*	*Lachnospiraceae NK4B4 group*	0.0	0.00021	**6.50E-05**	**0.0009**	5.56E-05	5.11E-05	0.55	0.54	0.037	0.052	**6.50E-05**	**0.0001**	Opposite time development
*Firmicutes*	*Lachnospiraceae UCG-001*	1.03E-05	0.00010	0.28	0.20	0.01930	0.01275	0.39	0.45	**3.95E-06**	**5.05E-05**	**1.89E-05**	**4.05E-05**	Increase during time development^c^
*Firmicutes*	*Lachnospiraceae UCG-004*	0.0	0.00029	0.060	0.074	0.00054	0.00051	0.62	0.57	**0.024**	**0.037**	**0.002**	**0.002**	Increase during time development^c^
*Firmicutes*	*Lachnospiraceae UCG-005*	0.0	4.88E-05	0.095	0.10	0.00034	0.00021	0.68	0.62	**0.005**	**0.012**	**0.003**	**0.003**	Increase during time development^c^
*Firmicutes*	*Lachnospiraceae UCG-006*	0.00139	0.00067	0.55	0.28	0.00247	0.00203	0.37	0.44	**0.013**	**0.023**	**0.004**	**0.004**	Increase during time development^c^
*Firmicutes*	*Lachnospiraceae UCG-010*	0.0	8.40E-05	**0.004**	**0.011**	2.12E-05	3.22E-05	0.79	0.66	**0.035**	**0.050**	0.063	0.034	Premature low level in PNGR
*Firmicutes*	*Lachnospiraceae Incertae Sedis*	0.00032	0.00247	0.044	0.065	0.00163	0.00143	0.70	0.63	0.39	0.31	**0.013**	**0.011**	Opposite time development
*Firmicutes*	*Lachnospiraceae [Uncultured]*	0.03506	0.070182	**0.002**	**0.007**	0.04509	0.05166	0.37	0.44	0.10	0.11	0.22	0.096	Premature low level in PNGR
*Firmicutes*	*Marvinbryantia*	0.00129	0.00198	0.23	0.17	0.006	0.00433	0.32	0.44	0.069	0.081	**9.53E-05**	**0.0002**	Increase during time development^b^
*Firmicutes*	*Roseburia*	0.00528	0.01254	0.062	0.074	0.00972	0.00987	0.91	0.70	0.48	0.35	**0.041**	**0.024**	Opposite time development
*Firmicutes*	*Ruminiclostridium 5*	0.00083	0.00190	**0.005**	**0.013**	0.00282	0.00364	0.31	0.44	0.051	0.068	**3.18E-06**	**1.13E-05**	Increase during time development^b^; Repressed at PN21 in PNGR
*Firmicutes*	*Ruminococcaceae NK4A214 group*	8.26E-05	1.83E-05	0.44	0.25	8.46E-05	0.000523	**0.0006**	**0.042**	**1.22E-05**	**8.84E-05**	0.99	0.31	No increase during time development in PNGR
*Firmicutes*	*Ruminococcaceae UCG-005*	0.0	0.0	0.95	0.40	0.0	0.00045	0.013	0.079	**4.35E-06**	**5.05E-05**	0.26	0.11	No increase during time development in PNGR
*Firmicutes*	*Ruminococcaceae UCG-010*	0.0	0.0	0.17	0.14	0.0	0.00028	0.017	0.087	**6.60E-05**	**0.0003**	0.90	0.29	No increase during time development in PNGR
*Firmicutes*	*Ruminococcaceae UCG-013*	0.0	0.0	0.35	0.21	0.0	0.00052	0.025	0.11	**0.001**	**0.004**	0.66	0.22	No increase during time development in PNGR
*Firmicutes*	*Ruminococcaceae UCG-014*	**0.00407**	**1.54E-05**	0.005	0.012	0.00220	0.00453	0.040	0.15	**0.011**	**0.021**	0.18	0.084	Opposite time development
*Firmicutes*	*Ruminococcus gauvreauii group*	0.0	2.83E-05	**0.004**	**0.011**	1.59E-05	0.0	0.082	0.21	**0.006**	**0.012**	**0.049**	**0.028**	Opposite time development
*Firmicutes*	*Staphylococcus*	0.00040	2.63E-05	**0.0002**	**0.002**	1.22E-05	1.85E-05	0.28	0.43	0.84	0.53	**1.89E-05**	**4.05E-05**	Premature high level in PNGR
*Firmicutes*	*Streptococcus*	0.00150	0.00028	**1.80E-05**	**0.0004**	0.00012	0.00012	1.0	0.72	**5.81E-05**	**0.0003**	**4.41E-10**	**1.41E-08**	Decrease during time development^c^; Remarkably high at PN21 in PNGR
*Firmicutes*	*Turicibacter*	0.0	0.0	0.15	0.14	0.0	3.16E-05	0.011	0.079	**5.04E-05**	**0.0003**	0.13	0.064	No increase during time development in PNGR
*Firmicutes*	*Tyzzerella*	0.00064	0.00086	0.54	0.28	9.44E-05	0.00011	0.78	0.66	0.084	0.096	**0.0007**	**0.0009**	Decrease during time development^b^
*Firmicutes*	*Tyzzerella 3*	0.0	0.0	0.089	0.096	0.0	0.00011	0.046	0.15	**0.002**	**0.005**	**0.023**	**0.016**	Increase during time development^c^
*Firmicutes*	*Clostridiales [Other]*	1.69E-05	1.47E-05	0.48	0.26	5.79E-05	4.07E-05	0.74	0.65	0.49	0.35	**0.014**	**0.011**	Increase during time development^b^
*Firmicutes*	*Firmicutes Family XIII [Eubacterium] brachy group*	0.00011	7.52E-05	0.96	0.40	0.00016	0.00012	0.30	0.44	0.46	0.35	**0.015**	**0.012**	Increase during time development^b^
*Firmicutes*	*Firmicutes Family XIII [Eubacterium] nodatum group*	7.88E-05	0.00015	0.77	0.35	0.0	0.00014	0.003	0.075	0.83	0.53	**0.019**	**0.014**	Decrease during time development in PNGR only
*Firmicutes*	*Firmicutes Family XIII [ClosFamily XIII AD3011 group*	0.0	0.0	0.56	0.28	4.81E-05	4.08E-05	0.55	0.54	**0.011**	**0.021**	**0.005**	**0.005**	Increase during time development^c^
*Firmicutes*	*Firmicutes Family XIII [ClosFamily XIII UCG-001*	2.63E-05	0.00022	**0.002**	**0.007**	0.00039	0.00049	0.36	0.44	**0.004**	**0.011**	**1.74E-06**	**9.33E-06**	Increase during time development^c^; Repressed at PN21 in PNGR
*Proteobacteria*	*Bilophila*	0.00013	0.00013	0.85	0.37	0.00022	0.00052	0.008	0.08	**0.001**	**0.004**	0.45	0.17	No increase during time development in PNGR
*Proteobacteria*	*Desulfovibrio*	0.01413	0.00781	0.14	0.14	0.00716	0.00978	0.10	0.22	0.91	0.57	**0.008**	**0.007**	Decrease during time development in PNGR only
*Proteobacteria*	*Escherichia-Shigella*	0.00221	5.42E-05	**0.008**	**0.017**	0.0	8.51E-06	0.084	0.21	0.055	0.070	**8.50E-06**	**2.27E-05**	Remarkably high at PN21 in PNGR
*Proteobacteria*	*Oryza meyeriana [mitochondrial]*	0.0	0.0	0.49	0.26	2.24E-05	0.0	0.10	0.22	0.94	0.57	**0.017**	**0.013**	Increase during time development in PNGR only
*Proteobacteria*	*Parasutterella*	0.00052	0.00061	0.27	0.19	0.00166	0.00205	0.28	0.43	**0.006**	**0.014**	**0.0002**	**0.0003**	Increase during time development^c^
*Tenericutes*	*Anaeroplasma*	1.31E-05	0.0004	0.073	0.083	0.00546	0.00232	0.025	0.11	**0.021**	**0.034**	**0.0002**	**0.0003**	Increase during time development^c^

Median relative abundance is reported for each genus in control (CTRL) and postnatal growth restriction group (PNGR) at postnatal day 21 (PN21) and postnatal day 60 (PN60). *P*-values < 0.05 and *Q*-values < 0.05 were regarded as significant. ^a^Time development significant in CTRL group only, but same direction of change in PNGR group; ^b^Time development significant in PNGR group only, but same direction of change in CTRL group; ^c^Time development significant and in same direction in both CTRL and PNGR groups. (CTRL, n = 18 from 6 litters; PNGR, n = 18 from 6 litters) (results are representative of three independent experiments).

### PNGR affects susceptibility to colitis in adult mice

In order to investigate whether PNGR had long-term impact on intestinal immune homeostasis, we induced chronic colitis by oral administration of 3% dextran sodium sulfate (DSS) in adult mice. CTRL and PNGR adult mice received drinking water (CTRL-H_2_O; PNGR-H_2_O) or 3% DSS in drinking water for three cycles consisting of five days of DSS followed by seven days of regular drinking water (CTRL-DSS; PNGR-DSS). Histological severity of the colitis, colon length and cytokines expression were evaluated at the end of the three cycles.

**Figure 5:**
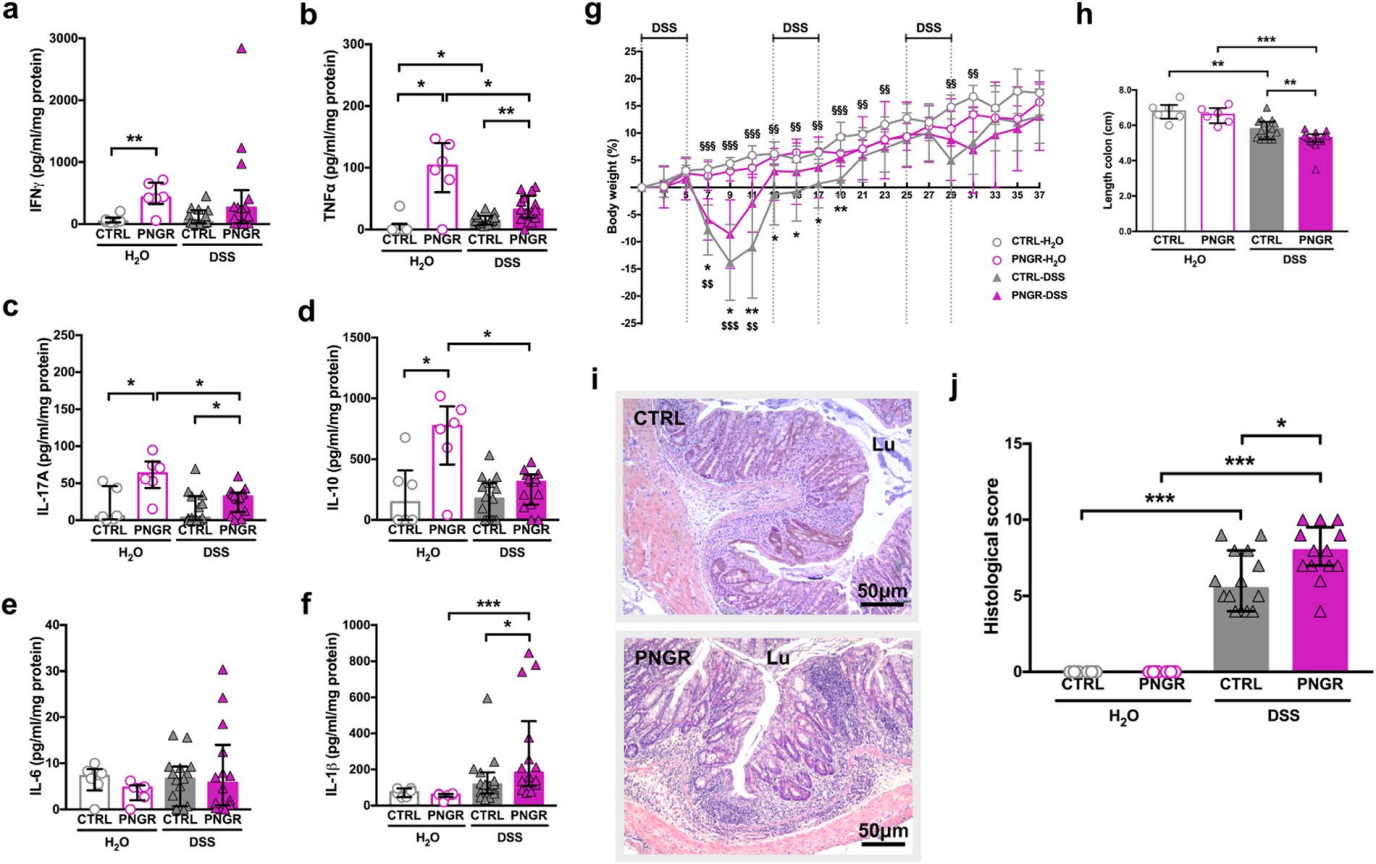
PNGR induced higher susceptibility to chronic chemically induced-colitis in adult mice. Control (CTRL) and postnatal growth restriction (PNGR) adult mice received drinking water (CTRL-H_2_O, n = 6 from 3 litters; PNGR-H_2_O, n = 6 from 3 litters) or 3% dextran sodium sulfate (DSS) in drinking water for three cycles consisting of five days of DSS followed by seven days of regular drinking water (CTRL-DSS, n = 19 from 6 litters; PNGR-DSS, n = 15 from 4 litters). **(a–f**) Cytokines expression in colon in CTRL and PNGR groups. **(g**) Body weight variation. *P < 0.05; **P < 0.01, PNGR-DSS vs. CTRL-DSS. ^$$^*P* < 0.01; ^$$$^*P* < 0.001, PNGR-DSS vs. PNGR-H_2_O. ^§§^*P* < 0.01; ^§§§^*P* < 0.001, CTRL-DSS vs. CTRL-H_2_O. **(h**) Colon length. **P < 0.01; ***P < 0.001. **(i**) Representative haematoxylin and eosin staining of colonic sections after the three cycles of DSS and **(j**) histological score of colitis severity. *P < 0.05; **P < 0.01; ***P < 0.001. *P < 0.05; ***P < 0.001. Results are representative of 2 independent experiments.

Adult PNGR mice not exposed to DSS (PNGR-H_2_O) expressed higher pro-inflammatory cytokine levels within the intestinal mucosa, indicating that PNGR resulted in an altered immune status in adult mice without prior DSS challenge. In ileum, the expression of the pro-inflammatory cytokine IL-1β was increased in adult mice with PNGR (*P* = 0.007, Suppl. Fig. S4a), as were the expression levels of the cytokines IL-10 (*P* = 0.004, Suppl. Fig. S4b) and IL-17 (*P* = 0.01, Suppl. Fig. S4c). IFN-γ, TNF-α and IL-6 levels were similar in both groups (Suppl. Fig. S4d–f). In colon, the expression of the Th1 cytokines IFN-γ (*P* = 0.009, Fig. 5a) and TNF-α (*P* = 0.02, Fig. 5b) was increased in adult mice with PNGR, as was the expression of the Th17 cytokine IL-17 (*P* = 0.02, Fig. 5c), and the regulatory immune response associated cytokine IL-10 (*P* = 0.02, Fig. 5d). The expression of the cytokine IL-6, associated with a Th2 immune response (*P* = 0.22, Fig. 5e), and IL-1β (*P* = 0.56, Fig. 5f), was similar in both groups.

DSS colitis induced significant body weight loss in both groups. The body weight loss was maximal on day 9 during the acute phase of the colitis and was less pronounced in PNGR group (*P* = 0.03, Fig. 5g). After day 9, the animals in both groups recovered reaching their baseline body weight by day 25 in control group and by day 13 in PNGR group (Fig. 5g). Chronic administration of DSS induced a more pronounced decrease in colon length in PNGR group (*P* = 0.006, Fig. 5h). The histological severity score was higher for PNGR mice (*P* = 0.02, Fig. 5i,j). After the three cycles of DSS, TNF-α was the only cytokine that was increased in the colon in CTRL group (CTRL-H_2_O vs. CTRL-DSS: *P* = 0.02, Fig. 5b). The colonic expression of the pro-inflammatory cytokines TNF-α, IL-1β, and IL-17 was higher in PNGR mice than in CTRL (CTRL-DSS vs. PNGR-DSS: *P* = 0.006, Fig. 5b; *P* = 0.04, Fig. 5f and *P* = 0.04, Fig. 5c, respectively). The expression of the regulatory immune response associated cytokine IL-10 decreased in the PNGR group after the three cycles of DSS (PNGR-H_2_O vs. PNGR-DSS: *P* = 0.01, Fig. 5d). The increased histological severity score, the more pronounced shortening of the colon, the persistent increased expression of pro-inflammatory cytokines, and the decrease in regulatory immune response suggest a higher susceptibility to chronic chemically induced-colitis in PNGR adult mice.

## Discussion

In the present study we first determined whether early life nutrition could impact intestinal maturation. We observed that PNGR altered the maturation of the intestinal barrier in pups at weaning, characterized by a reduction in ileal and colonic crypt depth, a thinner colonic mucosa thickness, a reduction in colonic goblet cells and an impaired expression of tight junction proteins. These morphological changes were associated with an increased intestinal permeability. The differences observed in expression and activity of DPPIV and IAP reflect probably a delay in functional maturation of the ileum in PNGR group. The expression of the digestive enzymes at the brush border appears with the differentiation of enterocytes during the third week of life in rodents^18–20^, and is associated with a decrease of the digestive enzymes activity^21,22^. The alteration of the intestinal maturation induced by PNGR is consistent with previous experimental studies testing the effects of nutritional interventions in early life^10,11,23–25^. While these studies are extremely diverse regarding the nutritional interventions and intervention windows, they have consistently concluded that inappropriate early life nutritional environment have deleterious impact on intestinal morphogenesis.

Secondly, we demonstrate that PNGR affected the bacterial colonization of the gut. PNGR induced a decrease in microbiota richness in pups, and led to lower abundance of *Odoribacter* spp. and Lachnospiraceae members, and higher abundance of *Parabacteroides* spp., *Erysipelatoclostridium* spp., *Eubacterium coprostanoligenes* spp., *Enterococcus* spp., *Staphylococcus* spp., and *Escherichia-Shigella* spp. at weaning. The genera *Odoribacter* spp. and Lachnospiraceae contain bacterial species known to be butyrate producers^26^. Butyrate is a short chain fatty acid produced by bacterial fermentation, and is the most important energy source for intestinal epithelial cells^27^. Butyrate promotes proliferation and differentiation of Treg cells^28,29^, and tight junction assembly^30^, thereby enhancing the integrity of the intestinal barrier. We hypothesize that the dysfunction of the intestinal barrier observed at PN21 in PNGR group could be explained by a lower abundance in butyrate producers. Quantification of butyrate in fecal samples is required to confirm this hypothesis. Intestinal inflammation has been shown to increase intra luminal oxygen, promoting the growth of facultative anaerobic bacteria, such as *Enterococcus* spp., *Staphylococcus* spp. and *Escherichia-Shigella* spp^31^. Speculating and building on this, *Escherichia-Shigella* spp., an Enterobacteriaceae member, is known to induce pro-inflammatory response via the activation of Toll-like receptor-4 (TLR-4)^6,32^. The higher abundance of potential pro-inflammatory bacteria (i.e. *Enterococcus* spp., *Staphylococcus* spp. and *Escherichia-Shigella* spp.) in pups with PNGR is consistent with the increase in intestinal pro-inflammatory cytokines observed at weaning in this group. However, the demonstration of the induction of TLR-4 is necessary to confirm the role of the higher abundance of *Enterococcus* spp., *Staphylococcus* spp. and *Escherichia-Shigella* spp. in the intestinal pro-inflammatory response we observed.

We observed that PNGR resulted in an altered intestinal immune status in adult mice. Cytokine levels within the intestinal mucosa in PNGR adult mice indicated a pro-inflammatory state already without DSS challenge. DSS administration resulted in a body weight loss in both CTRL and PNGR groups confirming the efficacy of the induction of chronic colitis by DSS^33,34^. This body weight loss was more pronounced in CTRL group after the first cycle of DSS suggesting an exacerbated response to the DSS compared to the PNGR group during the acute phase of the colitis^35^. It is known that both acute and chronic DSS colitis reduce fat mass^36^. The difference in body composition (lower fat mass and higher lean mass in adult PNGR mice) could explain the lower body weight loss in PGNR group during acute DSS colitis. However, we didn’t compare the body composition of CTRL and PNGR mice during the DSS colitis to confirm this hypothesis. The preexisting intestinal pro-inflammatory state in PNGR group could explain that adult PNGR mice were better prepared to the acute colitis leading to a less severe response reflecting by their lower body weight loss during the acute phase of the colitis. However, intestinal mucosal healing to the long lasting chronic relapsing colitis induced by chronic administration of DSS was lower in PNGR group, reflected by the persistent inflammatory response in PNGR group at the end of the three cycle of DSS characterized by increased histological severity score, shortened colonic length and persistent increased expression of the pro-inflammatory cytokines TNF-α, IL-1β, and IL-17 compared to CTRL group. The early alteration of the intestinal barrier and the dysbiosis observed in pups with PNGR could have contributed to the observed higher severity for chronic colitis in later life. This contrasts with previously published models of neonatal deprivation where susceptibility to colitis in adulthood where associated with persistent lower body weight and increased intestinal permeability^12,13^. Recent data in mice have demonstrated that the microbiota shapes the development of the intestinal immune system. The intestinal permeability is increased during the first two weeks of life in rodents^37–39^, which is associated with translocation of commensal bacteria from the intestinal microbiota to mesenteric lymph nodes. The immature immune system is therefore challenged with bacterial antigens. This contributes to the education of the immune system^37,40^, and coins the later mucosal immune response and homeostasis. Studies in germ free mice have shown that bacterial colonization promotes the development of mesenteric lymph nodes and Peyer’s patches, as well as the proliferation and differentiation of immune cells, and regulates the balance between pro-inflammatory T cells and regulatory T cells depending on the bacterial species that colonize the gut^41^. For instance, Segmented filamentous bacteria induce Th17 cells differentiation, leading to a pro-inflammatory immune response^42^, whereas *Bacteroides fragilis* promote the differentiation of CD4^+^ T cells into IL-10 Treg cells^43^. The impact of the intestinal microbiota on the maturation of the immune system depends on the timing of colonization. Colonization of germ free mice with conventional microbiota before weaning but not in adulthood protects against the expansion of pro-inflammatory natural killer T cells^44^. While no more differences in microbiota and intestinal permeability were observed in adulthood, adult mice with PNGR harbored higher pro-inflammatory cytokine levels within the intestinal mucosa and an increased susceptibility to chronic colitis, suggesting a programming effect of the early life dysbiosis and alteration of the intestinal barrier on later life intestinal immune response. Even if the dysbiosis and increased intestinal permeability induced by suboptimal early life nutrition appear to be transient, they occur during the key period of maturation of the intestinal barrier and immune system and may have long-term impact on the intestinal immune homeostasis.

In conclusion, this study supports a programming effect of early life undernutrition on the health status and susceptibility to chronic intestinal disease in later life, and hence unlocks the potential of improved nutrition in early life to reduce the risk for developing gut disease in adulthood. While the underlying mechanisms are not yet fully understood, our results suggest a key role for the interplay between the intestinal barrier and the microbiota in early life in the proper immune education and later life intestinal immune homeostasis, and thereby potentially in the development of inflammatory bowel disease in later life.

## Methods

### Animals

All animal experiments were in accordance with the French Guidelines for the Care and Use of Laboratory Animals following the EU-Directive on protection of animals used for scientific purposes, and were approved by the Animal Care Ethics Committee of the Nord-Pas-de-Calais region (approval ID: 01564.01). Eight-week-old females and males FVB/NRj mice (Janvier Labs, Le Genest-Saint-Isle, France) were maintained under standard conditions in a specific pathogen-free environment, with a standard chow diet appropriate for breeding (RM3, Special Diets Services, France) ad libitum. After eight days of acclimation, female mice were mated (2:1) during eight days with FVB/NRj male breeders. During the whole gestation and lactation period, pregnant dams were fed a standard chow diet ad libitum. In order to eliminate as much as possible the impact of the prenatal period on lactation of the mothers and nutritional status of the pups^45^, litters were adjusted to eight pups per mother at PN1, and litters less than six or more than 11 pups were excluded to normalize lactation of each mother. At PN4, all pups were randomly distributed among mothers to generate large litters with 15 pups per mother to induce PNGR. Normal litters were maintained with eight pups per mother and served as CTRL group. Each pup from either normal or large litters was assigned to a new mother when litter size was manipulated to normalize social stress. After weaning at PN21, litters received a standard chow diet (RM3, Special Diets Services, France) ad libitum.

### Pup growth and body composition

Body weight of pups was measured every two days from PN4 to PN21 and weekly after weaning until PN60 using an analytical balance. Body weight gain between weaning (PN23) and adulthood (PN60) was calculated using the following formula: (body weight at PN60 - body weight at PN23)/body weight at PN23 × 100. Body composition of males from CTRL and PNGR groups was compared at PN60 using LaTheta 100 × -ray Computed Tomography scanner. Animals were anesthetized by intraperitoneal injection of ketamine and xylazine. Fifty slices per mice were made between shoulders and pelvis. Slices were analyzed using LaTheta software for quantification of the total fat mass, subcutaneous fat mass, visceral fat mass and lean mass.

### Tissue collection

Males were killed at PN21 or PN60 by cervical dislocation. Distal ileum and distal colon were excised. Tissues were immediately fixed in 4% paraformaldehyde (USB, 19943) for 18 h and paraffin embedded for histological and immunohistochemistry analysis or were snap frozen in liquid nitrogen and stored at −80 °C for subsequent analysis. For digestive enzyme activity assays, samples from ileum were opened longitudinally and rinsed with ice-cold saline (154 mM NaCl, 0.1 mM PMSF, pH 7.4). Ileal mucosa was then collected by scraping the luminal surface, and snap frozen in liquid nitrogen. Fresh feces were snap frozen in liquid nitrogen and stored at −80 °C for subsequent analysis.

### Histology

Haematoxylin and eosin (HE) staining and Alcian blue – periodic acid Schiff (AB-PAS) staining were performed on six-micron-thick paraffin embedded sections. Mucosa thickness, crypt depth and villus height were blindly measured in 10 well-oriented crypts or villi per section from three colonic and ileal segments per animal. The number of goblet cells was determined as described before^46^ from three colonic and ileal segments per animal.

### Immunohistochemistry

Six-micron-thick paraffin embedded sections were prepared as described before^46^. Sections were then incubated with primary antibody diluted in PBS/1% BSA overnight at 4 °C: anti-claudin7 polyclonal antibody (1:150; Invitrogen, 34–9100), anti-occludin polyclonal antibody (1:200; Novusbio, NBP1-87402), anti-IAP polyclonal antibody (1:500; Abcam, ab7322), anti-DPPIV polyclonal antibody (1:500; gift from A. Hubbard). For claudin7, occludin, and DPPIV immunostaining, a heat-mediated antigen retrieval pre-treatment was performed in sodium citrate buffer (10 mM sodium citrate, 0.05% Tween 20, pH 6.0) at 100 °C for 20 min and then at room temperature for 20 min. After three washes in PBS, sections were incubated with FITC-conjugated secondary antibody (1:150; Jackson ImmunoResearch, 111-096-046) in PBS/1% BSA for two hours at room temperature. Immunofluorescence using the lectins MAA, which recognizes the epitope of sialic acid α-2,3-galactose, and UEA1, which recognizes the epitope of fucose α-1,2-galactose, was performed as described before^46^. Sections were counterstained with Hoechst 33258 (1:1,000; Molecular probes, H3569) in PBS, and mounted with Mowiol mounting medium. Images from immunostaining were acquired using a Leica TCS LCSM (Leica Microsystems, InC., Exton, PA). Immunofluorescence quantification was blindly performed with ImageJ software 1.48 in 10 well-oriented crypts or villi per section from three colonic and ileal segments per mouse, and was expressed relative to the area of the crypt or the villus.

### FITC-dextran intestinal permeability assay

To assess *in vivo* intestinal paracellular permeability, mice were orogastrically gavaged with 4 kDa FITC–dextran (440 mg/kg body weight in PBS; Sigma-Aldrich, FD4) at PN21 or PN60. After four hours, blood was collected by cardiac puncture, and serum was isolated by centrifugation (1,000 × *g* for 10 min). Serum FITC-dextran concentration was determined by fluorometry at 485 nm using FLUOstar Omega microplate reader (BMG Labtech, Ortenberg, Germany).

### Digestive enzyme activity assays

Intestinal brush border was prepared by Mg^2+^ precipitation according to a method adapted from Fan *et al*.^19^. Mucosal scrapings were homogenized in ice-cold homogenizing buffer (50 mM D-Mannitol, 0.1 mM PMSF, pH 7.4 adjusted with 0.5 M HEPES buffer) using a Polytron homogenizer. Homogenate was centrifuged at 2,000 × *g* for 15 min at 4 °C. Supernatant was incubated with 100 μL of 100 mM MgCl_2_ for 15 min, and then centrifuged at 2,400 × *g* for 15 min at 4 °C. After removing the top foamy layer, supernatant was centrifuged at 19,000 × *g* for 30 min at 4 °C. Supernatant was discarded and brush border pellet was suspended in 500 μL of 300 mM D-Mannitol (pH 7.4 adjusted with 0.5 M HEPES buffer) and centrifuged at 30,000 × *g* for 30 minutes at 4 °C. Supernatant was discarded and final brush border pellet was suspended again in 500 μL of D-Mannitol buffer. Protein concentration was determined using the Pierce BCA Protein Assay Kit (Biotechnology, 23227). For IAP activity assay, brush border homogenates (10 μg protein) were incubated with 1 mM 4-nitrophenyl phosphate (Sigma-Aldrich, 73737), 2 mM potassium fluoride, and 4 mM MgCl_2_, in a final volume of 1 mL at pH 10.5 and 37 °C for 10 min. Enzyme reaction was stopped by adding 1 mL of 0.5 M NaOH. The end product of enzyme reaction, p-nitrophenol, was determined at 400 nm using FLUOstar Omega microplate reader (BMG Labtech, Ortenberg, Germany). For DPPIV activity assay, brush border homogenates (20 μg protein) were incubated with 1 mM Gly-Pro p-nitroanilide (Sigma-Aldrich, G2901) and 50 mM Tris-HCl in a final volume of 200 μL at pH 8.0 and 37 °C for 30 min. Enzyme reaction was stopped by adding 800 μL of 1 M acetate sodium (pH 4.5). The end product of enzyme reaction, 4-nitroaniline, was determined at 405 nm using FLUOstar Omega microplate reader (BMG Labtech, Ortenberg, Germany). Enzyme activity was expressed as international units per gram of protein. One unit corresponds to the hydrolysis of 1 μmol of substrate per minute at 37 °C.

### Chemically-induced colitis

Colitis was induced in adult mice (PN60) by oral administration of 3% DSS (TdB Consultancy AB, DB001) in drinking water for three cycles, each consisting of five days of DSS followed by seven days of regular drinking water for recovery. Body weight change was monitored every two days. Mice were sacrificed at the end of the three cycles. Histological severity of the colitis was blindly graded according to Dieleman *et al*.^47^. Cytokine expression was quantified on colonic samples.

### Cytokine expression

Colonic and ileal samples were homogenized in 700 μL of 0.1 M Dithiotreitol, 0.01% NP40, and Complete mini EDTA-free (Roche, 11836170001), and then centrifuged at 1,000 × *g* for 10 min. Concentrations of IL-1β (eBioscience, 88-7013-88), TNF-α (eBioscience, 88-7324-88), IL-17A (eBioscience, 88-7371-88), IL-6 (eBioscience, 88-7064-88), IFN-γ (eBioscience, 88-7314-88), and IL-10 (eBioscience, 88-7105-88) were determined in homogenized colonic and ileal samples using respective enzyme-linked immunosorbent assay (ELISA) kits according to the manufacturer’s instructions.

### Statistical analysis

Variables were expressed by median and interquartile range. The non-parametric Wilcoxon-Mann-Whitney test was used to compare unpaired data. Comparison between groups and time-points of the data related to DSS treatment was performed using the Kruskal-Wallis test followed by a Mann-Whitney test. A *P*-value < 0.05 was considered significant. Statistical analysis was performed using GraphPad Prism® 7.0 (GraphPad Software, Inc).

### Microbiota analysis

Bacterial DNA extraction from fecal samples was performed with QIAmp DNA Stool Mini Kit® (Qiagen, 51504) according to the manufacturer’s protocol combined with bead-beating (FastPrep®-24 instrument, program 5.5) at lysis and homogenization steps. Extracted DNA purity was checked using the NanoDrop™ spectrophotometer (Thermo Fisher Scientific Inc.), whereas DNA quality and concentration were measured using the Qubit® dsDNA BR Assay kit (Life technologies, Q32850). DNA aliquots were stored at −80 °C for subsequent analysis. On the purified fecal DNA extracts primers Bact-0341F (5′-CCTACGGGNGGCWGCAG-3′) and Bact-0785R (5′-GACTACHVGGGTATCTAATCC-3′)^48^ were used to amplify the V3-V4 regions of the bacterial *16S rRNA* gene. The generated amplicons were subsequently sequenced on an Illumina MiSeq instrument as described previously^49^. Sequencing data was analyzed using the Quantitative Insights Into Microbial Ecology (QIIME) v.1.9.0 pipeline^50^. The quality filtered sequences were grouped into OTUs by de novo OTU picking using the USEARCH algorithm^51^ at 97% sequence identity. Subsequently, the Ribosomal Database Project Classifier (RDP)^52^ was applied to assign taxonomy to the representative sequence (i.e. the most abundant sequence) of each OTU by alignment to the SILVA ribosomal RNA database (release version 1.1.9)^53^. ChimeraSlayer^54^ was applied, as part of QIIME, to filter for chimeric sequences and these were excluded from all downstream analyses.

Weighted UniFrac distances were used to assess the (dis)similarities between the samples^55,56^. Rarefaction was applied to the OTUs by QIIME to ensure identical number of reads per sample in order to perform α-diversity calculations using the Chao1 metric. For the identification of the key bacterial groups (or taxa) that are associated to the different study groups, the relative abundance (expressed in proportion, where 1 = 100%) of each taxon was compared by using the Wilcoxon-Mann-Whitney test. The expected proportion of false positives, i.e., the false discovery rate (FDR), was estimated by calculating *Q*-values from the entire set of *P*-values (for all taxa) of a given comparison^57^. Resulting *P*-values < 0.05 with corresponding *Q*-values < 0.05 were regarded as significant.

## Supplementary information

Supplementary figures

## Data Availability

The dataset generated during this study is available from the corresponding author on request.
