# Supplementary material for: Early life nutrition influences susceptibility to chronic inflammatory colitis in later life
Source: Sci Rep. 2019 Dec 2;9:18111. doi: 10.1038/s41598-019-54308-6 (PMC6889478; doi:10.1038/s41598-019-54308-6)
Supplement: Supplementary file 1 — Supplementary figures [file 41598_2019_54308_MOESM1_ESM.docx]

**Title: Early life nutrition influences susceptibility to chronic inflammatory colitis in later life**

Delphine Ley^1*^, Jean-Luc Desseyn^1^, Valérie Gouyer^1^, Ségolène Plet^1^, Sebastian Tims^2^, Ingrid Renes^2^, Mona Mischke^2^, Frédéric Gottrand^1^

1. Univ. Lille, Inserm, CHU Lille, LIRIC UMR 995, F-59000 Lille, France

2. Danone Nutricia Research, Utrecht, the Netherlands

| **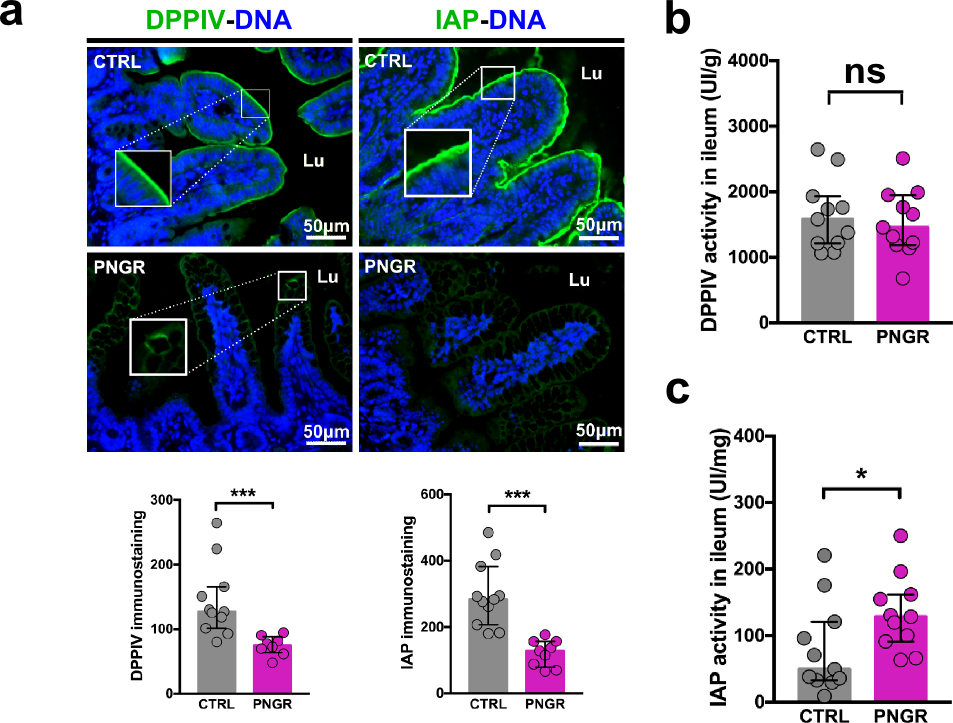** |
| --- |

**Supplemental figure S1**

**(a)** Representative immunostaining of dipeptidyl-peptidase IV (DPPIV) and intestinal alkaline phosphatase (IAP) in ileum at PN21 in CTRL (n = 11 from 4 litters) and PNGR (n = 8 from 4 litters) groups **(b)** DPPIV activity and **(c)** IAP activity in ileum at PN21 in CTRL (n = 11 from 4 litters) and PNGR (n = 11 from 4 litters) groups. **P*<0.05; ****P*<0.001.

| **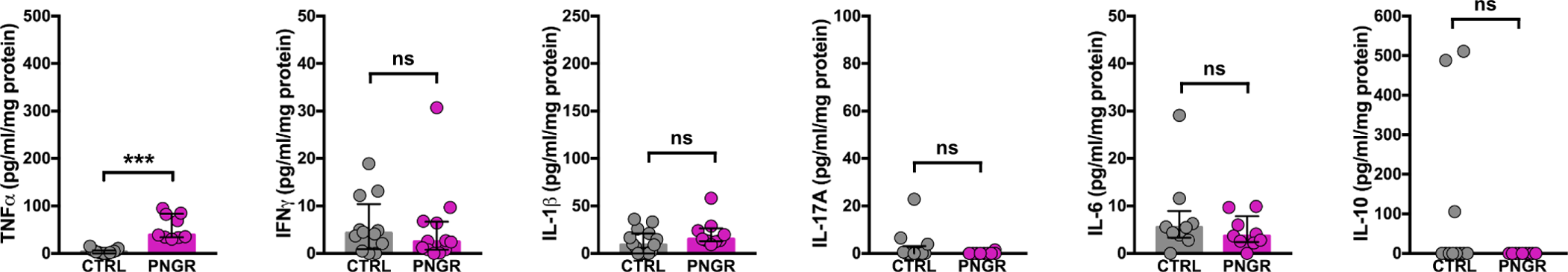** |
| --- |

**Supplemental figure S2**

Cytokines expression in ileum in control (CTRL, n = 9-15 from 4 litters) and postnatal growth restriction (PNGR, n = 9-12 from 4 litters) pups. Results are representative of two independent experiments. ****P*<0.001.

| **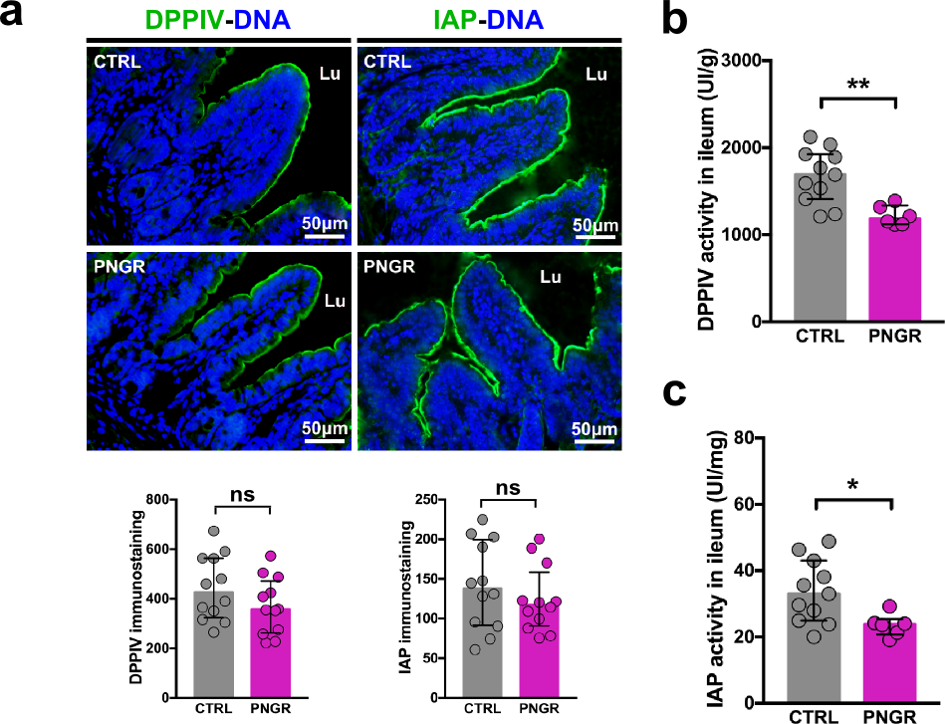** |
| --- |

**Supplemental figure S3**

**(a)** Representative immunostaining of dipeptidyl-peptidase IV (DPPIV) and intestinal alkaline phosphatase (IAP) in ileum at PN60 in CTRL (n = 12 from 4 litters) and PNGR (n = 12 from 4 litters) groups. **(b)** DPPIV activity and **(c)** IAP activity in ileum at PN60 in CTRL (n = 11 from 4 litters) and PNGR (n = 6 from 2 litters) groups. **P*<0.05; ***P*<0.01.

| **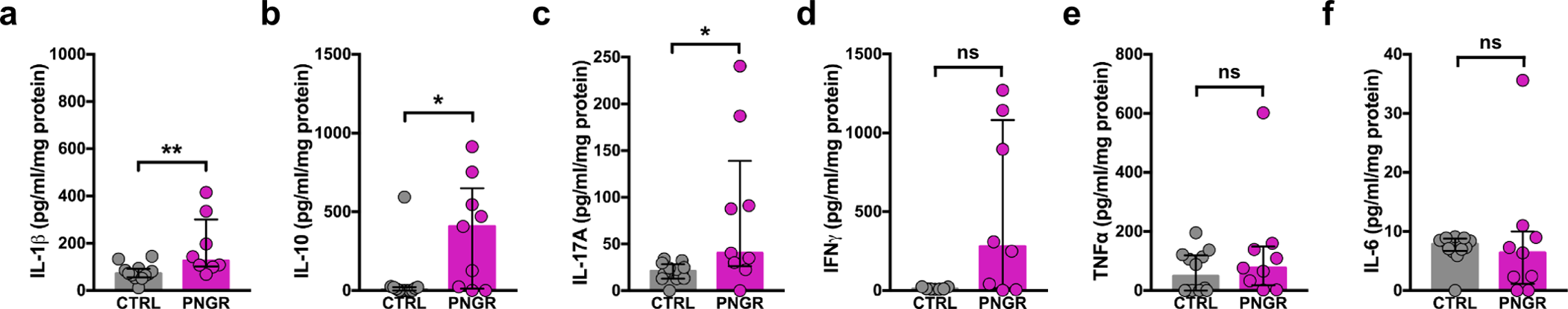** |
| --- |

**Supplemental figure S4**

**(a-f)** Cytokines expression in ileum in control (CTRL, n = 12 from 4 litters) and postnatal growth restriction (PNGR, n = 9 from 4 litters) adult mice. Results are representative of two independent experiments. **P*<0.05; ***P*<0.01.
